# Chronic Effects of a Dynamic Stretching and Core Stability Exercise Protocol on Physical Performance in U-16 Volleyball Players

**DOI:** 10.3390/sports13110413

**Published:** 2025-11-20

**Authors:** Annamaria Mancini, Loretta Francesca Cosco, Vincenzo Monda, Gian Pietro Emerenziani, Domenico Martone, Pasqualina Buono

**Affiliations:** 1 Department of Medical, Movement and Wellness Sciences, University Parthenope, 80133 Naples, Italy; annamaria.mancini@uniparthenope.it (A.M.); loretta.cosco@gmail.com (L.F.C.); pasqualina.buono@uniparthenope.it (P.B.); 2 CEINGE-Biotecnologie Avanzate “Franco Salvatore”, 80145 Naples, Italy; 3 Department of Economics, Law, Cybersecurity and Sport Sciences, University Parthenope, 80035 Naples, Italy; vincenzo.monda@uniparthenope.it; 4 Department of Clinical and Experimental Medicine, University Magna Graecia of Catanzaro, 88100 Catanzaro, Italy; emerenziani@unicz.it

**Keywords:** volleyball, dynamic stretching, core stability, performance, youth training

## Abstract

Background: Volleyball requires explosive jumps, agility, and upper and lower limb coordination. Dynamic stretching (DS) and core stability (CS) protocols are often used separately in training sessions, but little is known about their combined effects on the performance in adolescent players. This study aimed to investigate the impact of a 12-week integrated DS and CS program (StretCor), in addition to standard training, on physical performance in U-16 volleyball players. Methods: Twenty-one volunteer players (15.1 ± 0.6 years) were randomly assigned to the Intervention Group (IG; n = 12) or Control Group (CG; n = 9). IG performed the StretCor protocol four times a week for twelve weeks in addition to standard volleyball training; CG continued standard volleyball training. Physical performance assessment included Countermovement Jump (CMJ), Vertec jump with run-up, isometric shoulder strength (ASH-I), dynamic balance (mSEBT), and agility (*t*-test) tests. Results: Significant group × time interactions (*p* < 0.05, η^2^ ranged: 0.20–0.90) were found for CMJ height and peak power, Vertec jump, ASH-I, mSEBT scores, and *t*-test performance. Post hoc analyses showed improvements in IG for CMJ height (+16.5%), Vertec jump (+10.2%), shoulder strength (+11–14%), balance across directions (+8–12%), and agility (−5.7% *t*-test time). No significant changes were observed in CG. Conclusions: The present study suggests that a 12 weeks of StretCor protocol training improves jump performance, agility, dynamic balance, and upper limb strength in U-16 volleyball players. These findings also support that StretCor protocol may be beneficial for the performance when incorporated into regular training programs for adolescent athletes.

## 1. Introduction

Volleyball is characterized by numerous explosive actions involving the upper or lower limbs, such as wall jumping or spiking and serving which requires flexibility, power, balance, muscular strength, speed, and muscular endurance [1,2,3,4]. Moreover, a volleyball player during attacking and defensive phases needs to continuously change direction, speed, and body position, and perform accelerations and decelerations [5], which implies a good core stability (CS) to support the body stabilization and transmit energy to the limbs [6].

Stretching exercises are often used as strategies in different sports to improve the physical performance (PP) and reduce the injuries [7]. Among the different techniques used, dynamic stretching (DS), involving rhythmic movement-based exercises, promotes greater flexibility and muscle activation, enhancing neuromuscular preparation prior to athletic performance [8]. Unlike static stretching, which can transiently impair PP, DS has been shown to promote acute effects on athletic performance, such as sprint speed, agility, and jump height [9,10,11,12,13]. However, the chronic effects of DS on PP are still not completely understood. A systematic review analyzing 28 studies found that only 14 of them reported improvements in dynamic performance measures, such as jump height, sprint time, or other sport-specific abilities, particularly when the stretching interventions involved dynamic or ballistic elements [14]. This inconsistency in results may be attributed to several methodological differences across studies, including the type and intensity of dynamic stretching used, the duration and volume of the warm-up protocols, and the characteristics of the participants (e.g., age, training level). Such variability may mask potential performance benefits, suggesting the need for more standardized and sport-specific interventions.

In athletic conditioning, CS training has emerged as a fundamental component, particularly for sports involving multidirectional movements and explosive actions, such as volleyball. This type of training first enhances neuromuscular control of the lumbo-pelvic-hip complex, allowing for effective stabilization of the trunk during dynamic sport-specific tasks [8].

In volleyball, where powerful actions such as spiking, serving, and blocking require seamless high levels of coordination between the lower and upper limbs, trunk stability plays a crucial role in optimizing performance and reducing injury risk [15]. A recent comprehensive meta-analysis evaluating the chronic effects of core training on PP across different sports evidenced significant improvements in motor abilities, such as core endurance and balance, while effects on sport-specific outcomes such as sprinting, change in direction ability, and vertical jump performance were modest or non-significant [16]. However, in most of these studies [17,18,19], CS protocols were implemented in isolation alone, without integration with functional exercises, specifically deputed to promote coordinated activation of the segments involved in the kinetic chain during sport-specific movements. Furthermore, the effectiveness of core strengthening exercises depends on adequate spinal range of motion (ROM), particularly in movements involving flexion, extension, lateral flexion, rotation, and their combined patterns [1].

Generally, stretching and CS protocols are performed separately in the training session or on different days. Despite the potential benefits mediated by DS and CS training protocols separately, there is a lack of empirical evidence regarding the effects of training programs that combine both DS and CS training approaches specifically in adolescent volleyball players. In a previous study [20], we demonstrated that an acute session of combined DS and CS training improved lower limb muscle power, dynamic balance, and agility in U-16 volleyball players. Thus, we hypothesize that this integrated training protocol, carried out regularly in addition to traditional volleyball training, can improve PP components compared to standard volleyball training alone.

Therefore, the aim of the present study was to investigate the chronic effects of a 12-week StretCor training program, integrated into standard volleyball training, on key PP variables, including vertical jump height, dynamic balance, agility, and upper limb strength, in U-16 volleyball players.

## 2. Materials and Methods

### 2.1. Participants

Twenty-one volunteer athletes eleven males (age: 15.18 ± 0.66 years; body mass: 61.81 ± 10.99 kg; height: 1.64 ± 0.07 m; BMI: 22.93 ± 3.41 kg·m^−2^) and ten females (age: 15.07 ± 0.42 years; body mass: 60.60 ± 11.70 kg; height: 1.64 ± 0.08 m; BMI: 21.63 ± 2.87 kg·m^−2^), were recruited from the Volleyball Marcianise (Caserta, Italy) participating in the regional championship organized by the Campania Regional Committee of the Italian Volleyball Federation (FIPAV). Inclusion criteria: (i) age between 14 and 17 years; (ii) no musculoskeletal injuries in the three months prior to performance assessment; and (iii) a minimum of five years of experience in competitive sports. Exclusion criteria: any injuries that occurred in the three months prior to data collection. Informed consent was obtained from the participants’ parents or legal guardians, who were also informed that participants could withdraw from the study at any time or choose not to perform specific tasks included in the protocol. The study was conducted according to the Helsinki Declaration and approved by the regional ethics committee of University of Campania “Luigi Vanvitelli” (n. 0016488/i approved on 1 June 2023).

### 2.2. Study Design

**Figure 1:**
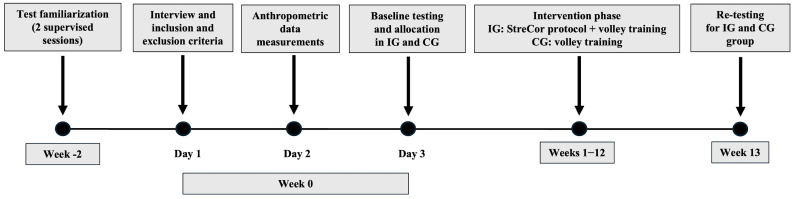
Timeline of the study design. IG, Intervention Group; CG, Control Group.

The study was conducted at the gym of the volleyball club where the athletes were recruited. In the first phase, on three non-consecutive days within one week, the participants did not engage in any training sessions or competitive matches (see timeline of the study design in Figure 1). On the first day, inclusion and exclusion criteria were verified for each participant through a structured interview. On the second day, anthropometric data, including body weight, height, and lower limb length, were collected. On the third day, following a standardized warm-up protocol (5 min jogging at a self-selected comfortable pace), participants completed a series of PP tests including: the modified Star Excursion Balance Test (mSEBT), Countermovement Jump (CMJ) and the Athletic Shoulder test I-position (ASH-I), Vertec (V) test with run-up approach, and agility *t*-test. After evaluation, participants were randomly allocated to either the Intervention Group (IG; n = 12; 5 males and 7 females) or the Control Group (CG n = 9; 6 males and 3 females) using simple randomization. A computer-generated random sequence was created by an investigator who was not involved in participant assessment or training supervision. Group allocation was revealed only after baseline assessments to ensure allocation concealment and reduce the potential risk of selection bias [21].

IG performed the StretCor protocol, in addition to specific volleyball training, four times a week for twelve weeks; the CG continued volleyball training alone. After the intervention period, all athletes belonging to both IG and CG were re-evaluated.

To ensure familiarity with the procedures, all participants underwent two supervised familiarization sessions two weeks prior to the start of data collection. Each test was explained in detail and performed under the supervision of qualified sport science professionals to minimize intra- and inter-rater variability. All assessments were conducted in the same indoor facility, under consistent environmental conditions (temperature: 22.1 ± 0.5 °C; relative humidity: 55 ± 2%) and at the same time of day (15:00–18:00) during the first part of the competitive season [22,23].

### 2.3. Testing Procedures

#### 2.3.1. Anthropometric Measures

Body mass and height were measured to the nearest 0.1 kg and 0.1 cm, respectively, using standardized equipment, including a stadiometer (SECA 213, Birmingham, UK) and an electronic scale (SECA 813, Birmingham, UK), with participants barefoot and wearing light clothing. Lower limb length was assessed in the supine position, measured from the anterior superior iliac spine to the midpoint of the ankle joint [24]. Additionally, the Moore equations [25] were applied to estimate the maturity offset, expressed as the number of years from peak height velocity (PHV), for each participant.

#### 2.3.2. Countermovement Jump (CMJ)

CMJ was performed as previously reported [26], using a ForceDecks Dual Force Plate System (Vald Performance, Brisbane, QLD, Australia). Participants began each trial from an upright standing position with their hands placed on their hips. They were instructed to perform a rapid downward movement by flexing the knees to approximately 90°, followed immediately by a maximal vertical jump. Each participant completed three trials, with one minute of passive recovery between attempts. The best trial for subsequent analysis was selected based on the highest jump height and relative peak power (W/kg). Jump height (cm) and relative peak power (W/kg) were automatically derived using the manufacturer’s proprietary software (Jump Application v2.0.8245, Vald Performance, Brisbane, QLD, Australia). In particular, jump height was derived using the impulse–momentum method: relative peak power was determined as the maximal power output during the jump divided by the participant’s body mass. Further methodological details regarding force–time metrics can be found in the Vald user manual (https://valdperformance.com/forcedecks/ accessed on 27 March 2025) and in previous research studies [27,28].

#### 2.3.3. Dynamic Balance Test (mSEBT)

Dynamic balance was assessed using the modified Star Excursion Balance Test (mSEBT) in three directions: anterior (ANT), posteromedial (PM), and posterolateral (PL). The layout of the testing grid and procedures followed the standardized protocol described previously [29]. Briefly, participants began the test standing barefoot with the most distal part of the great toe aligned at the intersection point of the Y-shaped grid, at the origin of the anterior line. While maintaining a single-leg stance on the support leg, participants were instructed to reach as far as possible with the contralateral leg along each of the three designated lines, lightly touch the tape with the distal part of the reaching foot, and then return the foot to the starting position without losing balance. Each participant performed three trials per direction for each leg. The test was initiated using the right leg as the stance leg, followed by the left, performing ANT, PM, and PL reach directions in sequence. A rest interval of 10 s was provided between trials within a direction. Six total attempts per direction were allowed to obtain three valid trials. The average of the three successful trials was used to calculate the normalized reach distance for each direction, expressed as a percentage of lower limb length (as measured previously). A composite score (COMP) was subsequently calculated by averaging the three normalized directional scores, in accordance with established procedures [30].

#### 2.3.4. Athletic Shoulder Test I-Position (ASH-I)

The Athletic Shoulder test (ASH), I-position, was administered to assess isometric force production across the shoulder girdle in the prone position, according to the protocol originally described by Ashworth et al. [31]. Briefly, participants lay prone on a force platform (ForceDecks Dual Force Plate System—Vald Performance, Brisbane, QLD, Australia) with their forehead supported on a 4 cm foam block. The testing arm was extended overhead (shoulder abduction ≈ 180°) with the palm in pronation and the elbow fully extended; the opposite arm remained comfortably by the side. Upon a verbal cue, participants were instructed to exert maximal isometric force into the plate as quickly as possible, maintaining peak contraction for 2–3 s, then relax and return to the starting position. Participants performed three consecutive trials with the right arm with a rest period of approximately 20 s between attempts to minimize fatigue effects and then three consecutive trials with the left arm, the best score was used for analysis.

#### 2.3.5. Vertec (V) Test with Run-Up Approach

In this test, athletes performed a maximal vertical jump following a run-up approach, replicating the typical take-off movement used during spiking actions. Before testing, standing reach height was measured with the athlete in a flat-footed position, reaching upward with the dominant hand to displace the highest vane on the Vertec device (JumpUSA Vertec, Sunnyvale, CA, USA). Participants were then instructed to execute a two- to three-step approach, followed by a rapid vertical jump using both arm swing and full-body coordination, aiming to touch the highest possible vane. The jump height was calculated as the difference between the standing reach and the highest vane touched. Each participant completed three trials, with at least one minute of rest between attempts, and the best score was retained for analysis. The Vertec test with run-up approach has demonstrated high ecological validity for volleyball performance and strong test–retest reliability (ICC > 0.90) when used to assess approach jump height [32].

#### 2.3.6. Agility *t*-Test

The agility test was performed according to the standardized procedure described by Semenick [33]. Participants completed the *t*-test by sprinting and shuffling between cones arranged in the shape of the letter “T”. Four cones were placed as follows: the starting cone (A), the central cone (B) positioned 9.14 m straight ahead from A, and two lateral cones (C and D) positioned 4.57 m to the left and right of B, respectively. Participants began each trial standing 50 cm behind the starting photocell sensor (Microgate, Bolzano, Italy), which was used to trigger the timing system. Upon initiating the movement, the photocell activated the connected stopwatch. Participants sprinted forward to cone B, touched it with the right hand, then shuffled left to cone C to touch it with the left hand, shuffled right past cone B to cone D to touch it with the right hand, shuffled back left to cone B and touched it again with the left hand, and finally backpedaled to the starting point (cone A), passing through the same photocell which stopped the timer automatically. Each participant performed three trials, with 30 s of passive recovery between attempts. The fastest trial time, recorded to the nearest one-hundredth of a second, was used for statistical analysis.

#### 2.3.7. StretCor Protocol

**Figure 2:**
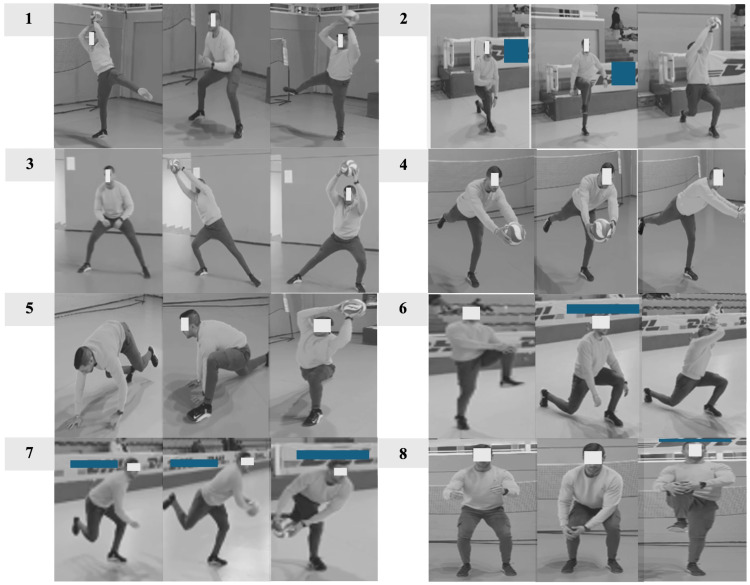
Numbered and illustrated sequence of the exercises included in the StretCor protocol. Adapted from: Martone et al. [20].

The StretCor protocol was designed to integrate dynamic flexibility and core stability activation in a brief, sport-specific sequence. It consisted of eight exercises performed in succession (seven out of eight with the use of the ball), with 15 s of passive recovery between each, for a total duration of approximately 10 min. The protocol targeted key muscle groups involved in volleyball-specific movements, including the hamstrings, hip flexors, shoulder girdle, and trunk musculature. Each exercise was selected to promote mobility, neuromuscular control, and functional stability in preparation for explosive and multidirectional actions. To provide a progressive overload stimulus, after starting using the volleyball ball for the first four weeks it was replaced with a medicine ball of 2 kg and 3 kg for male, and 1 kg and 2 kg for female athletes, during weeks 5–8 and 9–12 of the intervention protocol, respectively. The sequence visual illustrations and detailed descriptions of the StretCor protocol are provided in Figure 2; detailed descriptions of the exercises are provided in the Supplementary Materials File S1.

### 2.4. Statistical Analysis

Sample size was estimated using G*Power 3.1 based on the data from Martone et al. [20]. Assuming a Cohen’s d of 0.5, 80% power, and α = 0.05, a total of 18 participants (9 per group) was required to detect at least a 2% improvement in the performance variables.

Descriptive data are reported as mean ± standard deviation (SD). The Shapiro–Wilk test was used to assess the normality of distribution for each raw variable. All variables were checked for normality, and those that met the assumption of a normal distribution were analyzed using parametric tests, while non-normally distributed variables were analyzed using non-parametric tests. No data transformations were applied, as this approach appropriately accounts for non-normal distributions. The intraclass correlation coefficient (ICC) and coefficient of variation (CV) were calculated at baseline, using the repeated trials obtained during the familiarization/testing session, to determine the reliability and repeatability of the measurements before the intervention [34]. CV was calculated for each test at baseline by dividing the standard deviation of the repeated trials by the mean of those trials and multiplying by 100 (CV = [SD/mean] × 100). Participants were pooled together and randomly assigned to either the Intervention or Control Group using simple randomization. Although male and female participants were included in both groups, the study was not powered for sex-specific analyses. Therefore, all outcome variables were analyzed using pooled data. Between-group differences in anthropometric characteristics between participants were analyzed using either an unpaired *t*-test or the Wilcoxon–Mann–Whitney test, depending on data distribution. For each outcome variable, a two-way repeated-measures ANOVA (group × time) was performed to assess the main effects of group (intervention vs. control) and time (pre-test vs. post-test). When normality assumptions were violated, the Friedman test for within-group comparisons and the Mann–Whitney U test for between-group differences were used. Effect size (ES) was calculated by eta squared (η^2^). The magnitude of the difference was considered small (0.2), moderate (0.5), or large (0.8) [35]. When a significant interaction or main effect was detected, a correction for multiple comparisons was applied by a Bonferroni post hoc test to identify specific differences.

Jamovi software (version 2.3.28) was used for the analyses [36]. The significance level was set at *p* ≤ 0.05.

## 3. Results

The anthropometric characteristics of the volleyball players included in the study are summarized in Table 1.

No significant differences were observed between IG and CG for age and anthropometric variables (*p* > 0.05). All boys and girls were classified in the post-peak height velocity (PHV) phase, with maturity offsets of +1.30 ± 0.40 years and +2.70 ± 0.80 years, respectively. No significant difference in PHV was found between sexes (*p* > 0.05).

In Table 2 are summarized the parameters of reliability for the tests used. ICC value for jump height in CMJ and in V-test ranged from 0.927 to 1.010 and from 0.869 to 0.986, respectively; between 0.737 and 1.120 for peak power normalized to body mass (peak power/BM); from 0.927 to 1.030 and from 0.502 to 0.986 in right and left limbs, respectively, for ASH-I test; and from 0.54381 to 0.950 for the *t*-test scores. Further, for the mSEBT, ICCs for the right and left lower limbs across all reach directions, as well as for the composite (COMP) score, ranged from 0.757 to 0.980. Coefficients of variation (CVs) for all parameters ranged between 1.50% and 10.67%.

**Figure 3:**
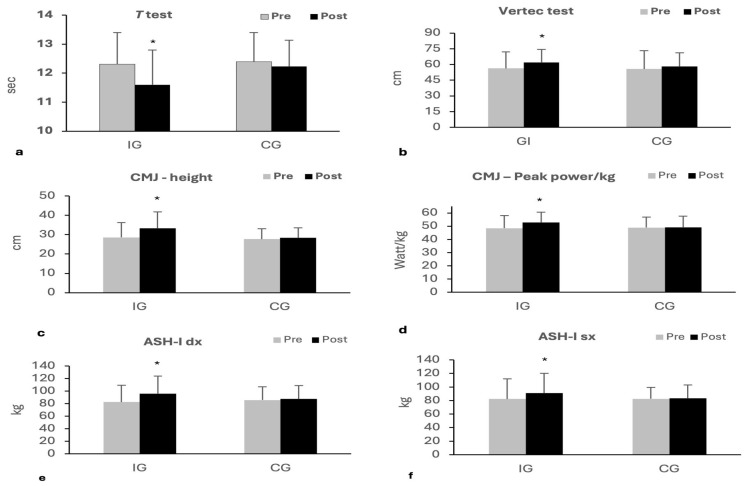
Mean ± standard deviation values for *t*-test (**a**), Vertec test (**b**), CMJ—height (cm) (**c**), CMJ—Peak power/kg (**d**), ASH-I dx (**e**), and ASH-I sx (**f**) in IG and CG in pre- and post-StretCor protocol training. CMJ, Countermovement Jump; ASH-I, Athletic Shoulder test version I. IG, Intervention Group; CG, Control Group. * Significant differences (*p* < 0.001) compared to pre-StretCor.

At baseline there were no significant differences between IG and CG in any variable measured. A significant time × group interaction (*F*_1,19_ = 28.2, *p* < 0.001, η^2^ = 0.60) and main effect for time (*F*_1,19_ = 2.2, *p* < 0.001, η^2^ = 0.79) were found for *t*-tests. Post hoc analysis showed a significant improvement in time performance in IG at post- compared to pre-intervention (11.60 ± 1.20 s vs. 12.31 ± 1.09 s, *p* < 0.001) while there were no differences in CG (12.39 ± 1.01 s vs. 12.23 ± 0.91 s, *p* = 0.318) (Figure 3a). The analysis of Vertec tests revealed a main time × group interaction (F_1,19_ = 5.2, *p* = 0.033, η^2^ = 0.22) and a main effect of time (F_1,19_ = 32.4, *p* < 0.001, η^2^ = 0.63). Post hoc tests indicated that IG has significantly improved the jump height at post- compared to pre-intervention (62.00 ± 17.63 cm vs. 56.25 ± 15.76 cm, *p* < 0.001); no changes were observed in CG (58.11 ± 13.03 vs. cm 55.67 ± 12.40 cm, *p* = 0.221; Figure 3b). CMJ tests showed a significant time × group interaction (*F*_1,19_ = 25.3, *p* < 0.001, η^2^ = 0.57) and main effect for time (*F*_1,19_ = 45.1, *p* < 0.001, η^2^ = 0.70) for jump height and a significant time × group interaction (*F*_1,19_ = 9.9, *p* = 0.005, η^2^ = 0.34) and main effect for time (*F*_1,19_ = 12.8, *p* = 0.002, η^2^ = 0.40) for relative peak power. Post hoc revealed a significant improvement in jump height and relative peak power in IG at post- compared to pre-intervention (33.30 ± 8.58 cm vs. 28.58 ± 7.62 cm, *p* < 0.001 and 52.91 ± 7.67 W/kg vs. 48.48 ± 9.48 W/kg, *p* < 0.001, respectively); no differences in CG (28.38 ± 5.13 cm vs. 27.70 ± 5.43 cm, *p* = 0.318 and 49.18 ± 8.51 cm vs. 48.90 ± 8.06 cm, *p* = 0.995, respectively) were observed (Figure 3c,d). The analysis of Athletic Shoulder tests revealed a time × group interaction (F_1,19_ = 29.5, *p* < 0.001, η^2^ = 0.60) and a main effect of time (F_1,19_ = 48.2, *p* < 0.001, η^2^ = 0.717) for right arm and a time × group interaction (F_1,19_ = 18.2, *p* < 0.001, η^2^ = 0.52) and a main effect of time (F_1,19_ = 23.5, *p* < 0.001, η^2^ = 0.55) for left arm. Post hoc tests indicated that IG significantly improved the isometric force production in both right and left arms at post- compared to pre-intervention (96.17 ± 27.96 kg vs. 82.50 ± 26.99 kg, *p* < 0.001 and 91.00 ± 29.18 kg vs. 82.25 ± 29.65 kg, *p* < 0.001, respectively); no changes were observed in CG (87.44 ± 21.56 kg vs. 85.78 ± 21.20 kg, *p* = 0.890 and 83.22 ± 19.81 kg vs. 82.67 ± 16.91 kg, *p* = 0.960, respectively) (Figure 3e,f).

The analysis of mSEBT tests revealed a significant time × group interaction (*p* < 0.05) and a main effect of time (*p* < 0.05) with ES ranging from small to large in all normalized reach distances in right and left lower limb. Post hoc analyses showed that IG significantly improved (*p* < 0.05) the percentage of normalized distance reached for each direction and COMPs in both right and left lower limb at post- compared to pre-intervention. No significant improvement (*p* > 0.05) was observed in CG (Table 3).

## 4. Discussion

The aim of the present study was to analyze the chronic effects induced by a combined training protocol including DS and CS functional exercises (StretCor) on some parameters related to the performance of a group of U-16 volleyball players. Our results showed that 12 weeks of StretCor protocol induced significant improvements in sport-specific physical parameters such as vertical jump height, relative peak power, dynamic balance, agility, and shoulder isometric force.

To the best of our knowledge this is the first study that evaluated the effects of an integrated training program, including exercises that stimulate both dynamic stretching and core stability, on physical capacities associated with performance in volleyball.

The results we obtained extend our previous observations of acute benefits of dynamic stretching and core activation exercise protocols in volleyball adolescent athletes [19] confirming that repeated exposure to an integrated program induces sustained adaptations relevant to sport-specific performance. Previous studies have separately considered the effects of training programs involving stretching or core exercises on different parameters associated with athletic performance. In particular, CS training enhances lumbo-pelvic control and improves the efficiency of force transfer from trunk to limbs, which is critical in explosive volleyball actions [15]. Şahin et al. [37] found that an 8-week core program improved vertical jump and balance in female volleyball players aged 12–14 years. Sharma et al. [38] observed that a 9-week trunk stability intervention enhanced spike and block jump performance, especially in athletes with low baseline trunk function. Similarly, the authors showed that 8 weeks of body-centering techniques into a core training program led to gains in explosive leg power and postural control in adolescent female volleyball players. Moreover, it has been reported that the gain in agility and vertical jump is improved when the exercises are sport-specific and functionally oriented and integrated into sport movement patterns [39].

The StretCor protocol integrated into the usual volleyball training produced greater improvements compared to the above mentioned studies [37,38] in jumping (CMJ: ∼+5%). Chronic DS interventions have been reported to increase muscle–tendon compliance, optimize use of the stretch–shortening cycle, and facilitate greater neuromuscular activation [10,40]. It may be hypothesized that DS combined with CS stimulus targeting both mobility and trunk stability may have enhanced neuromuscular adaptations which is particularly relevant for jump, balance, and agility tasks in volleyball as previously reported [11,40,41,42,43,44,45].

Upper limb strength adaptations are less commonly reported in volleyball, but indirect evidence is supportive. It has been reported that CS training enhanced throwing velocity and shoulder strength in female handball athletes [46], and serving accuracy improved following CS interventions in young volleyball players [6]. These findings are consistent with the hypothesis that trunk stabilization promotes proximal-to-distal force transmission during overhead actions [8]. Increased isometric shoulder strength observed in this study is in agreement with previous studies [6,46] supporting this hypothesis. Furthermore, the inclusion of DS may have induced additional benefits to shoulder function by maintaining range of motion and reducing stiffness, as previously reported [47], thereby complementing strength adaptations.

Taken together, the results obtained in this study suggest that protocols such as StretCor offer a more functional and comprehensive approach compared with traditional single-component training protocols. In fact, previous volleyball studies demonstrated improvements in isolated domains such as vertical jump [2,3], balance [4], or serving accuracy [5], whereas StretCor protocol, we proposed, provided concurrent gains in jumping, agility, balance, and shoulder strength. Thus, integrating StretCor protocol into the warm-up phase of standard volleyball training can provide a functional and time-efficient tool to improve multiple performance-related capacities in youth athletes. Coaches and practitioners may consider incorporating multi-joint, sport-specific exercises that target both mobility and trunk stability exercise to enhance jumping, agility, balance, and shoulder strength, all abilities critical for volleyball performance.

Some limitations must be acknowledged: (i) The relatively small sample size (n = 21) may result in overestimation of the observed effects due to increased variability and limited statistical power, potentially limiting the generalizability of this integrated protocol. On the other hand, the use of a homogeneous group minimized inter-individual variability, providing more reliable results. (ii) The lack of assessment of specific volleyball skills (e.g., serve accuracy and speed) prevented us from verifying the transferability of the performance gains obtained. (iii) Mechanistic variables such as electromyographic activity, tendon stiffness, or movement kinematics were not assessed, limiting insight into underlying physiological adaptations; IG accumulated a slightly greater total training time compared to CG due to the inclusion of ~10 min, each session, StretCor exercises in the standard volleyball training protocol. Although this increased volume (~10% on total volume) is an inherent component of the intervention being tested, it is possible that part of the performance enhancement is related to the supplementary workload itself. Future studies may consider including a time-matched placebo warm-up condition to fully isolate the specific effects induced by StretCor protocol.

## 5. Conclusions

The present study suggests that a 12-week program integrating dynamic stretching (DS) and core stability exercises (StretCor) performed during warm-up phase of standard volleyball training induced significant improvements in multiple domains of physical performance in U-16 volleyball players, including jump height, relative power, agility, dynamic balance, and isometric shoulder strength that are closely associated with sport-specific performance abilities in volleyball. These findings suggest that combining mobility and trunk stabilization strategies provides synergistic benefits that extend beyond those observed with isolated interventions. Further investigations are needed considering the long-term retention of adaptations induced by combined StretCor protocols during the competitive season, as well as exploring underlying neuromuscular mechanisms, assessing the transfer of gains to volleyball-specific skills. Moreover, it will be interesting to examine the applicability across different age groups, sexes, and competitive levels, which would further clarify the potential of these training strategies. Additionally, it would be interesting to examine how the variations in program volume, intensity, and exercise selection could help optimize protocols for youth volleyball players.

## Figures and Tables

**Table 1:** Anthropometric characteristics of participants (n = 21).

	IG (n = 12; 5 M, 7 F)	CG (n = 6 M; 3 F)	*p*-Value
Age (years)	15.2 ± 0.3	15.1 ± 0.8	0.782
Stature (m)	1.65 ± 0.1	1.63 ± 0.1	0.630
Body mass (kg)	61.9 ± 13.7	62.3 ± 8.0	0.943
Body mass index (kg·m^−2^)	22.7 ± 4.0	23.4 ± 2.6	0.640
Lower limb length right (cm)	86.0 ± 3.5	85.5 ± 5.4	0.801
Lower limb length left (cm)	85.8 ± 3.4	85.3 ± 5.4	0.800

IG, Intervention Group; CG, Control Group; M, males; F, females.

**Table 2:** Test–retest data for each variable assessed.

Test (Units)		ICC	95% CI	CV	95% CI
**mSEBT** normANT (%) normPM (%) normPL (%) COMP (%) **mSEBT** normANT (%) normPM (%) normPL (%) COMP (%) **Vertec (V) test** Jump height (cm) **ASH-I** MIF—right limb (kg) MIF—left limb (kg) **CMJ** Jump height (cm) Peak power/BM (W/kg) ***T*-test** (s)	Right lower limb Left lower limb	0.89 0.80 0.68 0.79 0.61 0.86 0.77 0.57 0.92 0.96 0.89 0.97 0.94 0.70	0.80–0.95 0.62–0.92 0.44–0.86 0.60–0.91 0.35–0.82 0.73–0.947 0.58–0.90 0.30–0.80 0.80–0.95 0.92–0.98 0.80–0.95 0.94–0.98 0.90–0.96 0.54–0.95	3.15 3.34 2.03 3.10 4.89 3.02 4.62 4.28 1.50 4.23 10.67 3.78 3.08 3.29	2.10–5.35 2.23–5.67 1.35–3.45 2.07–5.27 3.15–8.93 1.94–5.52 2.97–8.44 2.99–7.51 1.14–1.97 2.98–7.53 7.50–19.00 2.36–6.03 2.36–4.62 3.04–7.00

Note: mSEBT, modified Star Excursion Balance Test; normANT, normalized anterior direction; normPM, normalized posteromedial direction; normPL, normalized posterolateral direction; COMP, composite score; ASH-I, Athletic Shoulder test I-position; MIF, maximal isometric force; CMJ, Countermovement Jump; BM, body mass; ICC, intraclass coefficient correlation; CV, coefficient of variation; CI, confidence interval.

**Table 3:** Pre- and Post-StretCor protocol results of modified Star Excursion Balance Test (mSEBT) in IG and CG.

Variable (Units)	IG (n = 12)		CG (n = 9)		Main Effects	
	Pre	Post	Pre	Post	Time × Group	Time
**mSEBT** Right lower limb					F_(1,19)_	F_(1,19)_
normANT (%)	70.23 ± 8.48	77.16 ± 7.50 *	70.57 ± 4.87	70.53 ± 4.47	F = 30.2 (*p* < 0.001, η^2^ = 0.61)	F = 30.8 (*p* < 0.001, η^2^ = 0.61)
normPM (%)	91.62 ± 4.18	96.26 ± 4.20 *	95.12 ± 6.29	95.91 ± 6.44	F = 28.8 (*p* < 0.001, η^2^ = 0.62)	F = 57.2 (*p* < 0.001, η^2^ = 0.75)
normPL (%)	101.49 ± 12.37	106.43 ± 12.20 *	98.83 ± 7.46	99.83 ± 8.04	F = 34.2 (*p* < 0.001, η^2^ = 0.64)	F = 77.6 (*p* < 0.001, η^2^ = 0.80)
COMPs (%)	87.78 ± 7.48	93.28 ± 7.24 *	88.17 ± 3.58	88.61 ± 3.29	F = 78.0 (*p* < 0.001, η^2^ = 0.80)	F = 107.9 (*p* < 0.001, η^2^ = 0.85)
**mSEBT** Left lower limb					F_(1,19)_	F_(1,19)_
normANT (%)	70.26 ± 6.84	76.41 ± 7.64 *	69.89 ± 5.31	70.94 ± 5.89	F = 28.0 (*p* < 0.001, η^2^ = 0.59)	F = 56.1 (*p* < 0.001, η^2^ = 0.75)
normPM (%)	89.79 ± 4.82	94.13 ± 4.62 *	91.83 ± 6.15	93.51 ± 7.88	F = 6.3 (*p* < 0.001, η^2^ = 0.25)	F = 32.6 (*p* < 0.001, η^2^ = 0.63)
normPL (%)	97.89 ± 10.46	104.28 ± 11.64 *	96.53 ± 7.73	96.26 ± 8.75	F = 21.8 (*p* < 0.001, η^2^ = 0.53)	F = 18.4 (*p* < 0.001, η^2^ = 0.49)
COMPs (%)	85.99 ± 6.96	91.11 ± 7.32 *	86.08 ± 4.20	86.81 ± 4.61	F = 102.0 (*p* < 0.001, η^2^ = 0.84)	F = 191.0 (*p* < 0.001, η^2^ = 0.90)

Note: mSEBT, modified Star Excursion Balance Test; normANT, normalized anterior direction; normPM, normalized posteromedial direction; normPL, normalized posterolateral direction; COMPs, composite score. * Significant differences *(p* < 0.001) compared to pre. IG, Intervention Group; CG, Control Group.

## Data Availability

The data that support the findings of this study are available from the corresponding author upon reasonable request. The data are not publicly available due to privacy and ethical restrictions.

## Supplementary Materials

The following supporting information can be downloaded at: https://www.mdpi.com/article/10.3390/sports13110413/s1, File S1: Detailed description of StretCor exercise protocol.
