# Supplementary material for: Chronic Effects of a Dynamic Stretching and Core Stability Exercise Protocol on Physical Performance in U-16 Volleyball Players
Source: Sports (Basel). 2025 Nov 20;13(11):413. doi: 10.3390/sports13110413 (PMC12656671; doi:10.3390/sports13110413)
Supplement: Supplementary file 1 [file sports-13-00413-s001.zip › sports-3950385-supplementary.pdf]

Detailed description of StretCor exercise protocol

1. Sumo squat position, shift the weight of the body to the left lower limb, catch a ball thrown by a partner with the upper limbs extended, release the ball and return to the squat sumo position and repeat with the other limb. Repeat the exercise 20 times, 10 times for each limb.

2. Standing position hands at hips, move with the same limb in a consecutive manner, without placing the foot on the ground, first with the knee up towards the chest, then with a sideways rotation and then perform a rear lunge touching the ground with the knee. From this position take the ball thrown by a partner by extending upper limbs high above the head, then release the ball and from the rear lunge position, repeat the same with the left limb. Repeat the exercise covering the 9-metre lateral distance of the volleyball court twice.

3. Starting position with lower limbs spread wider than shoulder width upper limbs extended forward: perform two lateral lunges, first to the right and then to the left, until the knees are bent at 90 degrees. During the first lunge to the side, before moving on to the other lunge to the opposite side, a partner throws a ball, which must be caught with the upper limbs extended high above the head, then with the ball in hand perform the lunge to the opposite side and release the ball. Then turn with your body 180 degrees and perform two hops moving sideways and then resume the starting position and repeat the exercise again. Repeat the exercise covering the 9-metre lateral distance of the volleyball court twice.

4. Standing position, with a ball in the hands: extend right lower limb posteriorly and simultaneously bring your upper limbs forward with the ball in the hand, leaning your torso forward. Maintaining this position, first rotate your upper body to the right and then to the left, then return to the starting position. Rotate your body 180 degrees and repeat the exercise by extending your left lower limb. Repeat the exercise 20 times, 10 times for each limb.

5. Quadruped position with forefeet and hands on the ground: move forward with the right foot then holding on with the right and left foot extend the upper limbs up and catch a ball thrown by a partner and then release it and return with hands on the ground. Then pivoting with your right foot move forward with your hands on the ground (as spiderman style) and bring your left foot forward then holding onto your left and right foot extend your upper limbs up and catch a ball thrown by a partner and then release it and return with your hands on the ground. Repeat the exercise covering the 9-metre lateral distance of the volleyball court twice.

6. Standing position, bring the right knee with the hands until the chest, then release it and with the same leg lunge forward, then make a torso rotation to the left, bringing your upper limbs up, extending them and catching a ball thrown by a partner, then release it. On standing back up, bring the left knee with the hands until the chest, then release it and with the same leg lunge forward, then rotate your torso to the right, bringing your upper limbs up, extending them and catching a ball thrown by a partner, then release it. Repeat the exercise covering the 9-metre lateral distance of the volleyball court twice.
7. Standing position, in support with only the right left limb, right lower limb with flexed knee, right upper limb with flexed elbow and opposite the right knee and left upper limb extended behind. With the push on the right lower limb, perform a lateral and forward leap and move into the same starting position as the "speed skater" while catch the ball a ball thrown by a partner and then release it. Repeat the sequence covering the 9-metre lateral distance of the volleyball court twice.
8. Squat position, lower the upper body and bring your right lower limb by bending the knee and slowly bring it to your chest stretching the gluteus muscle, hold the position for two seconds and return to the starting position. Repeat the exercise 20 times, 10 times for each limb.
